# Physical education teachers’ attitudes toward inclusion of students with disabilities in Saudi Arabia

**DOI:** 10.3389/fpsyg.2022.1006461

**Published:** 2022-11-24

**Authors:** Majed M. Alhumaid, Bashaer A. Althikr Allah, Abeer A. Alhuwail, Maryam A. Alobaid, Naflah N. Abu Hamad, Zainab A. Alsalman, Sarah S. Alqahtani, Ayat M. Alherz, Walla M. Alwael, Aeshah K. Alhelal, Sheikh A. Alsubaie, Maryam S. Alwarthan, Fay O. Alnaeem, Shamma H. Aleid, Sara Y. Almuhaisen, Atheer A. Alobaydullah, Ameera R. Alzamami, Shuaa A. Alqadiri, Shoug H. Alsubhi, Abeer M. Alshikh, Khulud K. Almazrui, Madhawi A. Alamer, Afrah M. Alfadhel, Areej R. Al-Sari, Reamah S. Alqatari, Fatema A. Almaghrabi, Sara M. Alfahaid, Jailan A. Alhashim, Hawra A. Alsalman, Amnah A. Almatar, Najla M. Almutiri, Tânia Bastos

**Affiliations:** ^1^Department of Physical Education, College of Education, King Faisal University, Al-Ahsa, Saudi Arabia; ^2^Centre of Research, Education, Innovation, and Intervention in Sport, Faculty of Sport, University of Porto, Porto, Portugal

**Keywords:** inclusive physical education, inclusion, children with disabilities, physical educators, disability

## Abstract

The attitudes of physical education (PE) teachers toward inclusion are critical to the successful provision of inclusive teaching for students with disabilities. Therefore, the purpose of this study was to investigate the attitudes of Saudi Arabian PE teachers toward the inclusion of students with disabilities in PE classes and the effect of sociodemographic variables (e.g., gender and length of teaching experience) on their attitudes toward inclusion. A total of 1,314 PE teachers (*M*_age_ = 41.09, SD = 9.40, females = 42.8%) completed the Arabic version of the Sentiments, Attitudes, and Concerns about Inclusive Education-Revised Scale (SACIE-R). Analyses found that, in general, PE teachers had moderately positive attitudes toward the inclusion of students with disabilities in PE lessons. A significant difference was found between the two genders in their attitudes toward inclusion. Specifically, female PE teachers demonstrated more positive attitudes toward inclusion than males. Multiple linear regression analysis, meanwhile, showed that the length of teaching experience and the experience of teaching a student with a disability were significant predictors of participants’ attitudes toward inclusion. Our findings highlighted the importance of reconsidering the quality of PE teachers’ experiences and interactions with students with disabilities as a means to improving their attitudes, which in turn would translate into successful inclusion.

## Introduction

In the context of education, inclusion refers to the right of all students (regardless of their abilities) to be respected and included in the school community. However, achieving inclusion means more than simply implementing group-specific or disability-based approaches; inclusive policies seek to provide education that respects students’ dignity, equality, equity, justice, and human rights (San Martin et al., 2021).

In line with this principle, there is a growing trend in Saudi Arabia toward including students in the general system, regardless of their disability. This trend was triggered by legislation introduced in 2002 that emphasized the right of students with special needs to fully participate in school and extracurricular activities inside and outside of school and to have full access to school facilities. Teachers of students with disabilities must have a bachelor’s degree and a special education diploma or equivalent. Today, Saudi universities have more than 11 special education departments that train teachers in all aspects of disability. Non-governmental organizations and international organizations also play a crucial role in developing and expanding a basic education package, including counselors, principals, supervisors, and teachers. Such a policy is promising because empirical evidence has shown encouraging benefits for all students in terms of academic achievement and social skills (UNESCO, 2020).

Recent research also shows that the success of inclusion programs depends on teachers’ attitudes toward inclusion (Elhoweris and Alsheikh, 2006). Research on teachers’ attitudes reveals the use of a variety of terms with inconsistent theoretical references (Kunz et al., 2021). Attitudes can refer to the evaluative qualities of relationships to events and an individual’s responses to them (Eagly and Chaiken, 1993, p. 1), or they can relate to action-relevant setting constellations (Feyerer and Reibnegger, 2014). In the present study, though, teachers’ attitudes toward inclusive education refer to teachers’ opinions of or dispositions toward the particular “object” of inclusive education. The above attitudes may arise from beliefs about teaching students with disabilities in inclusive settings (i.e., the cognitive component of attitudes), feelings related to teaching those students (i.e., the affective component), and/or result from actions that favor their inclusion (i.e., the behavior component; Jury et al., 2021).

According to Forlin et al. (2011), a better understanding of teachers’ attitudes toward inclusion can help improve the learning environment (Ross-Hill, 2009). Educators with apprehensive attitudes may use practices that promote exclusion rather than inclusion in their classrooms (Sharma et al., 2008). On the other hand, educators who have positive attitudes toward inclusion tend to employ teaching strategies that allow them to accommodate individual differences (Campbell et al., 2003; Forlin, 2010). The inclusion of students with disabilities in mainstream schools and the construction of inclusive classrooms are likely to increase the number of diversity factors among students and help them overcome barriers to learning (Prast et al., 2018; Schwab et al., 2019).

According to Lindner et al. (2019), an inclusive approach to teaching children with disabilities may facilitate mutual attitudinal changes to promote better learning skills and a wide range of social motivations and practices. Therefore, teachers must adapt their teaching and learning practices to the specific needs of all students (Oliver and Oesterreich, 2013). In the context of physical education (PE) in particular, inclusion is by no means one-dimensional. Students with disabilities can benefit from social interactions, especially if these interactions are positive (e.g., supportive, collaborative, respectful), frequent, and meaningful, and if egalitarian relationships are encouraged (Qi and Ha, 2012). Block and Obrusnikova (2007) also stated in a literature review based on 38 English language research articles on inclusive physical education between 1995 and 2005 that students with disabilities can be successfully included in PE when adequate support is provided; moreover, this inclusion had no negative effect on non-disabled students.

Much previous research has also shown that, alongside the development of pedagogical skills and knowledge, positive teacher attitudes are crucial to the success of all students in inclusive classrooms (Bastos et al., 2017; Yada and Savolainen, 2017; Haegele et al., 2020; Saloviita, 2020). Teachers’ attitudes play an important role in forming students’ intentions to behave in positive ways (Braksiek, 2022). For example, teachers’ positive attitudes toward students not only encourage learners to be successful in their classes (Combs et al., 2010) and to develop both learning skills and knowledge of the subject matter but are also integral to successful inclusion (Yada and Savolainen, 2017; Saloviita, 2020). Consequently, to achieve successful inclusion in PE contexts, it is essential that PE teachers have positive attitudes and are adequately supported (UNESCO, 2020; Rojo-Ramos et al., 2022). Such support plays an important role in meeting Sustainable Development Goal 4 (UNESCO, 2016) in ensuring that all students have access to high-quality, inclusive, and equitable education and that opportunities are promoted for lifelong learning.

Unfortunately, however, it has been observed that many PE teachers experience stress and anxiety when working with children with disabilities. Teachers suffer from panic attacks because they fear that during a lesson with increased motor activity, they will harm the health of students or they will not be able to quickly and adequately adapt to disability-related difficulties (Catellani et al., 2018). The particular anxieties and psychological difficulties varied according to the type of disability, of which 35.8% were caused by working with children with autism spectrum disorder, 33.5% by working with children with intellectual disabilities, 32.1% by working with children with visual impairments, and 27.6% by working with children with hearing impairments. Only 52.3% of the participants were positive about the implementation of inclusive education, while 24.7% of them strongly opposed the inclusion of children with physical disabilities in educational events, 17.9% were indifferent to the implementation of inclusive education, and 5.1% remained silent. Moreover, only 38.9% of respondents confirmed the long-term effectiveness of inclusive education, 34.1% reported the ineffectiveness of inclusive education for all participants in the educational process, and 27% had difficulty evaluating the effectiveness of inclusive education at this stage of implementation (Sharifullina, 2011). Thus, there appears to be little consensus among PE teachers regarding the subject of inclusive education.

According to Avramidis and Norwich (2002), three main aspects (i.e., student characteristics, school conditions, and teacher characteristics) influence teachers’ attitudes toward inclusion. For example, male and female teachers have different attitudes toward teaching students with disabilities and hold different beliefs about inclusion. Several studies found that female teachers tend to report more positive attitudes toward inclusion (Papadopoulou et al., 2004; Vaz et al., 2015; Hutzler and Daniel-Shama, 2017). That said, Fournidou et al. (2011) found that male teachers reported significantly more positive attitudes toward inclusion than their female counterparts. These inconsistencies in the evidence suggest that the gender of teachers impacts their attitudes toward inclusion (Doulkeridou et al., 2011); however, Urton et al. (2015) demonstrated no gender impact on the same attitudes. In the Saudi context, meanwhile, there is a lack of research investigating the impact of gender on PE teachers’ attitudes toward inclusion because before 2018, there were no PE classes in girls’ schools in Saudi Arabia. Therefore, it is worth investigating the difference between Saudi males and females in their attitudes toward inclusion. Moreover, the educational qualifications of teachers appeared to impact their attitudes toward inclusion. For example, those with a master’s degree or higher exhibited more positive attitudes than those with a bachelor’s degree or lower (Ahmmed et al., 2012). However, the influence of “knowledge of intellectual disability” and “quality of contact (quality of experience in other studies) has been studied among 455 undergraduate students toward people with intellectual disabilities. In that study, Alnahdi (2019) reported that the combination of the two variables showed a significant influence on students’ attitudes toward people with intellectual disabilities. Neither knowledge about intellectual disabilities nor the quality of contact in people with intellectual disabilities alone significantly predicted attitudes toward people with intellectual disabilities.

The impacts of teachers’ ages and the length of their teaching experience have been widely investigated, but the results are inconsistent (Ginevra et al., 2021). Some studies (e.g., Tsakiridou and Polyzopoulou, 2014; Saloviita, 2020) revealed that younger and less experienced teachers tend to report more favorable attitudes toward inclusion than older and more experienced teachers, but other researchers, such as Markava et al. (2016), have disputed this, claiming that there is no association between teachers’ ages and the length of their teaching experience with their attitudes toward inclusion. In terms of the impact of teachers’ experiences of inclusive classes (either positive or negative), teachers who positively and successfully rated their experiences of teaching inclusive classes reported having more positive attitudes toward inclusion (Ahmmed et al., 2012). Moreover, according to Wilson et al. (2016), the ability of teachers to successfully apply inclusive teaching strategies depends on their own past performances.

Several studies have examined the extent to which having previous contact with students with disabilities influences teachers’ attitudes toward inclusive teaching practices. Providing persons without disabilities with opportunities to interact directly with people with disabilities positively affected the attitudes of the former toward the latter (McCarthy and Campbell, 1993; Meegan and MacPhail, 2006). Having teaching experience with people with disabilities and teaching students with disabilities also causes teachers to have more positive attitudes toward inclusion (Peebles and Mendaglio, 2014; Dorji et al., 2019). However, other studies (e.g., Tripp and Rizzo, 2006) found that previous contact with persons with disabilities did not influence individuals’ attitudes. Therefore, it is the quality, rather than the mere incidence, of such contact that is critical (Hutzler et al., 2019).

Thus, in order to determine whether a curriculum overhaul and additional teacher training are needed, it is necessary to conduct a study to examine the attitudes of PE teachers in Saudi Arabia toward the inclusion of students with disabilities in their PE classes and to identify which socio-demographic factors—including gender, age, education level, general experience, experience teaching students with disabilities, and benefits of adaptive physical education (APE) training—have the greatest impact on these teachers’ attitudes toward inclusion.

To achieve these goals, it is necessary to answer the following research questions:

1. Do Saudi PE teachers have a positive attitude toward the inclusion of students with disabilities in their PE classes?
2. Do male and female teachers have different attitudes toward including students with disabilities in their PE classes?
3. Do PE teachers differ significantly in their attitudes toward including students with disabilities in their PE courses by age, education level, general experience, experience teaching students with disabilities, and training in APE?
4. Which of the above factors significantly influenced the attitude of PE teachers toward the inclusion of students with disabilities in their PE classes?

## Materials and methods

### Participants

A total of 1,314 Saudi physical education teachers aged 24–59 years (41.09 ± 9.40), including 562 females and 752 males, teaching in private and public schools, completed an electronic questionnaire to assess their attitude toward including students with disabilities in physical education sessions (Forlin et al., 2011). The participants, who came from 13 regions in Saudi Arabia (Makkah: 20.4%; East: 19.6%; Najran: 13.7%; Riyadh: 8.3%; Asir: 8.1%; Qasim: 8%; Jazan: 5%; Madina: 3.9%; Albaha: 3.8%; others: 9.2%), volunteered to participate in this study and agreed to have their responses published publicly by checking the “Accept” box on the questionnaire.

### Instrument

For data collection, the Sentiments, Attitudes, and Concerns about Inclusive Education Revised (SACIE-R) scale was used. The SACIE-R consists of 15 statements designed to measure prospective teachers’ perceptions of three constructs of inclusive education, namely feelings or comfort in interacting with people with disabilities; acceptance of students with different needs; and concerns about the implementation of inclusion. Participants were asked to respond using a four-point Likert-type scale, with “strongly disagree, score 1″, “disagree, score 2″, “agree, score 3,” and “totally agree, score 4″. A mean score was calculated by summing all item scores and dividing by the total number of items. The higher a participant’s score, the more positive their attitude toward inclusive education for students with disabilities was deemed to be.

In addition to the 15 items used on the scale, the survey included a number of demographic questions relating to gender (male, female), age (25 and younger, 26–35, 36–45, 46+), academic degree in PE (diploma, bachelor, high diploma, postgraduate, none), length of teaching experience (0–5 years, 6–10 years, 11–15 years, 16–20 years, 21+), level of experience in teaching students with disabilities (nil, some, high [at least 30 full days]), and benefits of APE training (never helped me, helped me a little, helped me to a great extent, never studied this major). According to Forlin et al. (2011), the calculation of Cronbach’s alpha indicated good internal reliability for the 15-item of the scale (*α* = 0.74) and all three factors (*α* = 0.75, *α* = 0.67, *α* = 0.65 for sentiments, attitudes, and concerns, respectively). The SACIE-R instrument has been used in various countries, such as Bahrain (AlMahdi and Bukamal, 2019), Australia (McCracken et al., 2020), Ghana (Opoku et al., 2021), and Greece (Mouchritsa et al., 2022). As the participants in the current study spoke only Arabic, the SACIE-R was translated from English into Arabic by four bilingual (Arabic and English) special education and PE professors using the bilingual method (Brislin, 1970). The translation was based on the meaning of the English statements rather than a word-for-word translation. No items were removed during the translation process. The accuracy of the English and Arabic versions was then compared and any necessary changes were made. The construct validity of the Arabic version was assessed using exploratory factor analysis and principal component analysis, while its reliability was assessed using Cronbach’s alpha.

### Procedure

After obtaining ethical clearance from the research ethics committee at King Faisal University, Saudi Arabia (KFU-REC-2022-MAR-EA000478), PE teachers in Saudi Arabia were asked to participate in the study voluntarily. The questionnaire was sent by email with a link to an online questionnaire platform (Google Forms). By clicking on the link, the participants could read the information sheet, which contained the aims of the study and essential information. The received questionnaires (*N* = 1,452) were evaluated at the end of the data collection period, with a total of 138 questionnaires being removed due to non-completion. Therefore, 1,314 completed questionnaires were used for data analysis purposes.

### Statistical analysis

To answer the first research question, the mean (*M*) and the standard deviation (SD) of the attitude levels of Saudi PE teachers toward inclusion were calculated. For the purposes of research question two, the differences between the attitudes of male and female teachers toward inclusion were assessed using independent sample testing. One-way analysis of variance (ANOVA) was used to assess the significant differences between participants stratified by age, education level, general experience, experience teaching students with disabilities, and input from APE courses. To answer research question three, for variables where the null hypothesis was rejected, the Bonferroni post-hoc test was used to test for differences between subgroups. The linearity of predictor variables was tested using scatterplots, the normality and homoscedasticity of residuals were validated, and all independent variables were tested for multicollinearity using the variance of the inflation factor with values of less than 5. The multiple linear regression (MLR) analysis was used to answer the final research question. MLR analysis verified the age and sociodemographic variables’ associations with attitudes toward inclusion among all participants and across gender groups. The Statistical Package for the Social Sciences (SPSS) software (IBM SPSS Statistics 26.0. Ink, Chicago, IL, USA) was used to perform the statistical analysis, and the level of significance was set to *p* < 0.05.

## Results

### Reliability and validity of the Arabic version of the SACIE-R

Exploratory factor analysis (EFA) was used to identify the factorial validity of the items in the Arabic version of the SACIE-R. EFA was performed using the principal component analysis extraction approach based on the recommendations of Field (2009), followed by a varimax rotation to maximize the sum of variances. The Kaiser–Meyer–Olkin (KMO) sample fit measure was above the acceptable threshold of 0.6 (KMO = 0.758); Bartlett’s sphericity test result was statistically significant (*p* < 0.001). Three components with eigenvalues > 1 (3.596, 2.107, and 1.296) were identified and maintained; these accounted for 46.662% of the total variance, with weights >0.50 for all 15 items (corrected item-total correlation between 0.55 and 0.765), meaning that all items represented a three-dimensional construct with a significant contribution from all items.

The calculation of Cronbach’s alpha indicated good internal reliability for both the combined SACIE-R scale (*α* = 0.727) and all three factors (*α* = 0.793, *α* = 0.636, *α* = 0.611 for sentiments, attitudes, and concerns, respectively; Nunnally, 1978). Additionally, all items achieved an acceptable corrected item-total correlation of between 0.334 and 0.550 (Cristobal et al., 2007). Therefore, the Arabic version of the scale was found to be both reliable and valid for measuring the attitudes of Saudi PE teachers toward providing inclusive practices for students with disabilities.

### Demographic characteristics

The data indicated that 0.8% of participants taught kindergarten, 45% elementary school, 32.3% middle school, and 21.9% high school. Female teachers represented 42.8% of all participants. Most participants had a bachelor’s degree in PE (59.2%), while the remainder had a high diploma (7.5%), a postgraduate degree (5.9%), or did not hold a degree in PE (25%). Table 1 provides other relevant information on the teachers’ backgrounds and inclusion scores.

**Table 1:** Mean and standard deviation of attitudes toward inclusion among PE teachers stratified by gender, age, and sociodemographic variables.

		Males			Females			Total		
		*N*	Mean	SD	*N*	Mean	SD	*N*	Mean	SD
Gender		752	2.35	0.36	562	2.51	0.39	1,314	2.511	0.393
Age (years)	25 and less	21	2.37	0.41	32	2.48	0.46	53	2.44	0.44
	26–35	123	2.39	0.41	89	2.51	0.42	212	2.44	0.42
	36–45	223	2.34	0.34	293	2.50	0.37	516	2.43	0.36
	46+	385	2.33	0.36	148	2.52	0.41	533	2.38	0.39
Degree in physical education	Diploma	51	2.32	0.36	48	2.35	0.53	99	2.34	0.45
	Bachelor	607	2.36	0.37	171	2.53	0.39	778	2.40	0.38
	High Diploma	22	2.30	0.29	9	2.59	0.37	31	2.38	0.34
	Postgraduate	62	2.26	0.33	16	2.64	0.55	78	2.34	0.41
	None	10	2.15	0.39	318	2.51	0.36	328	2.50	0.36
Length of teaching experiences	0–5 years	84	2.35	0.41	463	2.53	0.36	547	2.50	0.38
	6–10 years	61	2.40	0.42	23	2.43	0.48	84	2.41	0.44
	11–15 years	75	2.42	0.38	24	2.43	0.36	99	2.42	0.37
	16–20 years	111	2.33	0.35	18	2.43	0.49	129	2.34	0.37
	21+	421	2.33	0.34	34	2.36	0.61	455	2.33	0.37
Level of experience of teaching a student with a disability	Nil	285	2.47	0.35	477	2.54	0.37	762	2.51	0.36
	Some	310	2.33	0.34	67	2.41	0.42	377	2.34	0.35
	High (at least 30 full days)	157	2.16	0.36	18	2.10	0.64	175	2.15	0.39
Benefits of Adapted Physical Education training	Never helped me	161	2.43	0.35	39	2.42	0.50	200	2.43	0.38
	Helped me little	304	2.33	0.36	46	2.48	0.38	350	2.35	0.36
	Helped me to a great extent	122	2.20	0.38	14	2.32	0.49	136	2.21	0.39
	Never studied this major	165	2.39	0.34	463	2.52	0.38	628	2.49	0.37

### The scores for attitude toward inclusion among physical education teachers

Overall, the results indicated that the attitudes toward inclusion among all participants were moderately positive (*M* = 2.511, SD = 0.393). Specifically, 4.57% of participants had low scores for their attitudes toward inclusion (score < 1.75), 52.66% had a moderate score (1.75 ≤ score < 2.5), 41.4% of participants’ scores were high (2.5 ≤ score < 3.25), and 1.37% of participants’ scores were very high (score ≥ 3.25). Moreover, independent sample T-tests indicated a significant difference between males and females in their attitudes toward the inclusion of students with disabilities in PE lessons (*t* = −7.795, *p* < 0.001); specifically, female PE teachers demonstrated a more positive attitude toward such inclusion (*M* = 2.51 and *M* = 2.35, respectively).

As can be seen in Table 2, the one-way ANOVA revealed a significantly more positive attitude toward inclusion among PE teachers with a bachelor’s degree compared with those with a diploma or a postgraduate degree in PE (*p* < 0.01 for all) as well as among PE teachers with little teaching experience (<5 years) compared with the two most experienced groups (16–21 years and 21 years +, *p* < 0.001 for both). Significant differences were noted between female teachers with a high-school diploma in PE and those with a bachelor’s degree (*p* < 0.05). Additionally, the scores for attitudes toward inclusion were significantly higher among participants with little or no experience in teaching students with disabilities than in those with high levels of experience (*p* < 0.01 for female teachers with some experience, *p* < 0.001 for others). A significant difference was also found between participants with no experience and those with some experience of teaching students with disabilities (*p* < 0.05 for female teachers, *p* < 0.001 for male teachers). The score for attitudes toward inclusion was lower among male teachers who reported that studying for a PE degree at university had prepared them to a greater extent than in the other groups of male teachers.

**Table 2:** Differences in PE teachers’ attitudes toward inclusion.

		Total (*N* = 1,314)		Males (*N* = 752)		Females (*N* = 562)	
		*F*	*p*	*F*	*p*	*F*	*p*
Age (years)	25 and less	*No significant differences*					
	26–35						
	36–45						
	46+						
Degree in physical education	Diploma	6.108	0.001	*No significant differences*		2.592	0.038
	Bachelor						
	High Diploma						
	Postgraduate						
	None						
Length of teaching experiences	0–5 years	14.56	0.001	*No significant differences*			
	6–10 years						
	11–15 years						
	16–20 years						
	21+						
Level of experience of teaching a student with a disability	Nil	79.959	0.001	41.283	0.001	13.607	0.001
	Some						
	High (at least 30 full days)						
Benefits of Adapted Physical Education training	Never helped me	25.017	0.001	10.96	0.001	*No significant differences*	
	Helped me a little						
	Helped me to a great extent						
	Never studied this major						

### Predictors of attitudes toward inclusion

Table 3 illustrates the importance, direction, and strength of the associations between predictor variables and attitudes toward the inclusion of students with disabilities in PE classes among Saudi PE teachers (stratified by gender). In all participants, the length of teaching experience and level of experience of teaching a student with a disability were predictors, with negative values for both (*β* = 0.02 and *p* < 0.05) and (*β* = 0.156 and *p* < 0.001) standardized beta values, respectively; these results indicate that PE teachers with less teaching experience and PE teachers with low levels of experience in teaching a student with a disability are more likely to have more positive attitudes toward inclusion. The same predictors were also found for female teachers, albeit with weaker effects (*R*^2^ = 0.053 versus 0.117 for the overall sample). However, for male teachers, the benefits of APE training (*β* = 0.029, *p* < 0.05) was the second-strongest predictor of the level of inclusion among the participants; this suggests that those who claimed that studying APE prepared them the most had lower scores for inclusion. The experience of teaching a student with a disability was the most important and strongest contributor to the level of inclusion for all participants and in all teacher subgroups (Table 3).

**Table 3:** Multiple linear regression analysis for the associations between attitudes toward inclusion and age, and sociodemographic variables among PE teachers, stratified by gender.

			Unstandardized coefficients		*t*	Sig.	95.0% confidence interval for *B*	
		*R* Square	*B*	Std. error			Lower bound	Upper bound
Total (*N* = 1,314)	(Constant)	0.117	2.026	0.069	29.326	<0.001	1.891	2.162
	Length of Teaching Experiences		−0.02	0.010	−1.985	0.047	−0.039	<0.001
	Level of experience teaching a student with a disability		−0.156	0.016	−9.816	0.001	−0.125	0.187
Males (*N* = 752)	(Constant)	0.108	2.148	0.080	26.689	0.001	1.990	2.306
	Level of experience of teaching a student with a disability		−0.152	0.017	−8.907	0.001	−0.119	0.186
	Benefits of Adapted Physical Education training		−0.029	0.012	−2.407	0.016	−0.052	−0.005
Females (*N* = 562)	(Constant)	0.053	2.061	0.124	16.572	<0.001	1.817	2.305
	Length of Teaching Experiences		−0.034	0.016	−2.185	0.029	−0.065	−0.003
	Level of experience teaching a student with a disability		−0.158	0.037	−4.316	0.001	−0.086	0.230

Regression β coefficients (unstandardized) represent the degree of change in the inclusion score for every 1-unit of change in the predictor variable.

## Discussion

The current study’s first aim was to investigate the attitudes of Saudi Arabian PE teachers toward the inclusion of students with disabilities in PE classes, while its second aim was to examine the effect of sociodemographic variables (e.g., gender and length of teaching experience) on their attitudes toward inclusion. There are several related studies in this context mainly conducted in North and South America, Europe, Australia, and East Asia (Hutzler et al., 2019). To the best of our knowledge, this is the first study to address inclusive education among Saudi male and female PE teachers—although other researchers have investigated teachers’ attitudes toward inclusion in general education contexts in Saudi public schools (e.g., Alshahrani, 2014; Alasim and Paul, 2019; Yada and Alnahdi, 2021) and private schools (e.g., Aldosari, 2022).

The findings of this study demonstrated that participants held moderately positive attitudes toward the inclusion of students with disabilities in their PE lessons. This result contradicts that of Aldosari (2022), who reported a slightly unfavorable general attitude toward the inclusion of students with disabilities among general education teachers in private elementary schools in Riyadh (Saudi Arabia’s capital). Our conclusion also differs from that of Yada and Alnahdi (2021), who reported that Saudi teachers did not express extreme attitudes for or against inclusive education. Neutral attitudes have also been noted among Saudi teachers toward the inclusion of hearing-impaired students (Alasim and Paul, 2019) and learning difficulties (Al-Ahmadi, 2009). In the international context, several studies (e.g., Doulkeridou et al., 2011; Majoko, 2019; Emmers et al., 2020) have reported that teachers exhibited positive attitudes toward inclusive practices. The finding is also supported by a more recent study conducted by San Martin et al. (2021) who found that teachers were more likely to have positive attitudes toward inclusion. However, the current study’s finding is inconsistent with research (e.g., Hersman and Hodge, 2010; Rekaa et al., 2019) that has demonstrated that PE teachers have negative attitudes toward the inclusion of students with disabilities in their classes. Therefore, more research is required to explore the factors that affect (positively or negatively) PE teachers’ attitudes toward inclusion.

In regards to the second objective, the current study’s findings revealed a significant difference between males and females. Specifically, the female participants reported more positive attitudes toward the inclusion of students with disabilities than their male counterparts. This finding is in line with that of Alasim and Paul (2019), who reported that Saudi female teachers have more positive attitudes toward the inclusion of hearing-impaired students compared to their male counterparts. However, it differs from those of Al-Ahmadi (2009) and Alquraini (2011) who reported that male teachers exhibited more positive attitudes toward inclusion in general compared to their female counterparts. According to Alasim and Paul (2019), these differences can be attributed to differences in the types of students’ disabilities and the variability of methods and strategies used in pre-service preparation programs for female teachers compared to those used for male teachers. As male and female Saudi teachers are trained in separate educational programs due to religious and cultural principles, initial female teacher training programs may focus more on developing positive attitudes toward inclusion compared to those for males.

The current study’s findings are also inconsistent with a recent Saudi investigation that indicated that male PE teachers demonstrated higher levels of confidence toward the inclusion of students with autism than female participants (Alhumaid, 2021). One possible explanation for why the current study’s findings are inconsistent with Alhumaid’s (Alhumaid, 2021) findings might be the difficulties that PE teachers face when including students with autism in their PE classes. For instance, Lindblom et al. (2020) reported that teachers’ feelings were negative toward the inclusion of students with autism in their classes. Because of the linguistic and cognitive difficulties faced by students with autism, teachers must take on additional challenges when including such students in their classes (Beamer and Yun, 2014). Therefore, more research is required to clarify the real attitudes of Saudi PE teachers toward the inclusion of students with different types of disabilities. Another explanation as to why female participants in the current study reported more positive attitudes toward inclusion than their male counterparts could be that the former are willing to attend more courses and have experiences related to inclusive practices (Rojo-Ramos et al., 2022), which may be reflected in their attitudes and feelings toward teaching students with disabilities in their classes.

The current study also found that teachers’ educational level positively influences their attitudes toward inclusion. Research has shown that being more highly educated leads to greater knowledge and skills across a wider range of teaching methods (Muñoz and Chang, 2007). This additional experience, in turn, can lead teachers to discover that they are better equipped to meet the needs of all students regardless of disability status (Mngo and Mngo, 2018). However, contrary to our conclusion, Alquraini (2012), Alasim and Paul (2019), and more recently Aldosari (2022) did not find that educational level had a significant impact on Saudi teachers’ attitudes toward inclusion. The lack of educational diversity among most participants in the above-mentioned studies provides a possible explanation for this non-significant effect of education level on Saudi teachers’ attitudes toward inclusion (Aldosari, 2022). Our finding contradicts also that of Ruppar et al. (2016), who found that teachers with postgraduate degrees displayed more confidence in including students with disabilities than those with bachelor’s degrees. However, San Martin et al.’s (San Martin et al., 2021) study found no significant difference in teachers’ attitudes in terms of their academic degrees. Therefore, more research into the effect of academic degrees and their impact on teachers’ attitudes toward inclusion is required.

The findings on the predictors of all the participants’ attitudes toward the inclusion of students with disabilities suggested that the length of teaching experience (the frequency of contact in other studies) and the level of teaching students with disabilities were negatively significant predictors. The length of teaching experience negatively predicted participants’ attitudes toward the inclusion of students with disabilities in PE classes. Participants with less experience exhibited more positive attitudes toward inclusion compared with those with more experience.

The results of this study differ from those of Alnahdi et al. (2020), who reported that the quality of contact, rather than its duration, was found to be the most influential factor in attitudes toward people with disabilities. Meaningful, close, and intimate contact with people with disabilities is necessary. These authors also suggested that early intervention would be necessary for contact to improve not only in quantity but also in quality. These interventions should also include increased knowledge of disability (Alnahdi, 2019). However, our results confirm those of Alasim and Paul (2019) who reported that having previous experience teaching students with disabilities is unrelated to attitudes toward inclusion. In other words, the attitudes of teachers who have experience teaching students with disabilities appear to be comparable with those of teachers without such experience. Similarly, studies by Savolainen et al. (2012) and Yada and Savolainen (2017) have identified that attitudes toward inclusion among teachers with high levels of teaching experience are negative. One explanation as to why less experienced teachers demonstrated more positive attitudes toward inclusion than those with more teaching experience could be that the former has received more training on teaching students with disabilities and on inclusiveness (Hutzler et al., 2019). It could also be because, recently, many countries have enacted legislation and policies and adapted their education systems to adopt inclusion in schools (Granell et al., 2021). Therefore, teachers with more teaching experience (i.e., older teachers) need more awareness training to help them keep abreast of such developments.

Interestingly, the level of experience of teaching students with disabilities (i.e., the second significant predictor of participants’ attitudes toward inclusion) was a negative predictor of participants’ attitudes. In particular, participants who reported having low levels of experience in teaching students with disabilities were more likely to have more positive attitudes toward the inclusion of students with disabilities than those with high experience levels. It is possible that participants’ previous experiences of teaching students with disabilities in the current study were not successful, which may be reflected in their attitudes toward inclusion. This finding coincides with a previous study that found that interacting with people with disabilities may not influence attitudes (Tripp and Rizzo, 2006). Moreover, it has been found that the quality of teachers’ experience with people with disabilities plays a critical role in their attitudes toward inclusion (McManus et al., 2011; Ahmmed et al., 2012; Keith et al., 2015), i.e., teachers with previous successful experiences of teaching students with disabilities are more likely to have positive attitudes toward inclusion. Wilson et al. (2016) indicated that teachers’ past performances in implementing inclusive practices help them to continue implementing such practices successfully.

Nevertheless, staff development through continuous professional development and fostering collaborative organizational cultures remains a fundamental practice of effective school management (Maciver et al., 2018). Shared experiences can provide opportunities to engage community members by building relationships, empowering each participant, creating an identity as an inclusive classroom or school, and visibly engaging all members of the learning community. Research on the cultural aspect of inclusive education has shown how, through the process of co-creation, community members highlight the essential elements of inclusion and thus build and strengthen the culture of inclusion in their school. The transparency promoted in the process of developing a more inclusive school culture allows the elements of inclusion to be exercised, thus strengthening the inclusion process (McMaster, 2013, 2014).

Although the current study has certain strengths, it must also be acknowledged that it suffers from some limitations, each of which needs to be discussed and addressed in future research. First, the values recorded and analyzed were based only on self-reported responses to the questionnaire. Therefore, the possibility of recall bias and social desirability outcomes cannot be ruled out. Second, the study’s target population included all individuals responsible for teaching PE to kindergarten, primary, middle, and high school students. The authors knew in advance that not all respondents, especially females, were PE teachers (do not have a degree in PE) and that many of them had no specific training in PE. In fact, it was not until 2017 that PE classes for girls became an integral part of the Saudi education system. Prior to this date, PE was exclusively for boys and not taught at all to girls, and indeed it is still not widely taught to girls to this day. Third, although the current study asked about the participant’s level of experience teaching students with disabilities, it did not ask about their feelings or evaluations of those experiences. The favorable attitudes of PE teachers are linked to earlier successes in involving students with disabilities (Ahmmed et al., 2012). Finally, because the current study used the Arabic version of the SACIE-R to collect self-reported data from participants, it was unable to obtain empirical data on the participants’ feelings or behaviors in their PE lessons. Consequently, direct and indirect observations may be effective ways to assess PE teachers’ attitudes toward the inclusion of students with disabilities. In addition, the use of cross-sectional data does not allow for the study of causal relationships between variables, and, therefore, future longitudinal studies are needed.

## Conclusion

To conclude, it is important to highlight the several strengths of this study, to emphasize the implications of the findings for the inclusive policies of Saudi Arabia, as well as to identify the future necessary steps to make Saudi Arabian schools truly inclusive.

Regarding the strengths of the study, we would like to highlight that data collection was developed at the national level, which meant we were able to ensure substantial geographical representativeness across different regions of the country. Likewise, data collection crossed all teaching levels where PE is taught, from kindergarten to high school. Moreover, to the best of our knowledge, this is the first study conducted in Saudi Arabia including female participants, meaning this is the first time that the attitudes of women PE teachers toward inclusion have been explored. This is because until very recently a PE degree was not accessible to women and, consequently, there were no female PE teachers in the country.

The present study also strived to clarify the contribution of specific variables, such as a degree in PE or length of teaching experience, to explain the attitudes of PE teachers toward inclusion. These variables have already been explored in previous studies but several inconsistencies remained that needed to be clarified and predictive statistical approaches have not always been used to provide more robust evidence. In our opinion, the findings of this study call for deep reflection. Around 25% of the participants did not hold a diploma, which means that a high number of children at schools are being educated by people that lack proper training and qualifications for the roles and responsibilities assumed. It is thus urgent that educational policies are improved in order to guarantee that all professionals working at schools receive proper certification. Moreover, in the present study, the participants with higher qualifications in PE and with more preparedness for teaching were those with lower attitude scores. These are very concerning findings that highlight the need to assess the quality and appropriateness of adapted physical activity/education courses included in the training of future PE teachers in Saudi Arabia.

Finally, it is important to stress that educating through positive examples is a critical mission for societies in general and educational settings in particular. Inclusive schools are also a setting where both male and female teachers should be valued and represented with equity. This is an important tool to empower girls with disabilities to be successful in life and to promote positive attitudes toward people with disabilities of all genders. Therefore, educational policies in Saudi Arabia must embody inclusive values and principles from a broader perspective to ensure that equity and diversity are respected across all minorities and vulnerable groups. Furthermore, this vision of inclusion should not only focus on the socio-economic factors that influence teachers’ attitudes toward inclusion but also on the changes that schools need to make in behaviors, environments, routines, and staff structures (Allan, 2012). These ideas align with concepts in disability theory, particularly the social model of disability, which rejects attention to individual impairment and focuses on the disabling aspects of culture and institutions (Oliver, 2013).

## Data availability statement

The raw data supporting the conclusions of this article will be made available by the authors, without undue reservation.

## Ethics statement

The studies involving human participants were reviewed and approved by Research Ethics Committee at King Faisal University, Saudi Arabia (KFU-REC-2022-MAR-EA000478). The patients/participants provided their written informed consent to participate in this study.

## Author contributions

MA was the project manager. All authors listed have made a substantial, direct, and intellectual contribution to the work and approved it for publication.

## Funding

This research was funded by the Deanship of Scientific Research, Vice Presidency for Graduate Studies and Scientific Research, King Faisal University, Saudi Arabia, grant number GRANT482. The funder had no role in the design of the study; in the collection, analysis, or interpretation of data; in the writing of the manuscript, or in the decision to publish the results.

## Conflict of interest

The authors declare that the research was conducted in the absence of any commercial or financial relationships that could be construed as a potential conflict of interest.

## Publisher’s note

All claims expressed in this article are solely those of the authors and do not necessarily represent those of their affiliated organizations, or those of the publisher, the editors and the reviewers. Any product that may be evaluated in this article, or claim that may be made by its manufacturer, is not guaranteed or endorsed by the publisher.
